# Four-way classification of Alzheimer’s disease using deep Siamese convolutional neural network with triplet-loss function

**DOI:** 10.1186/s40708-023-00184-w

**Published:** 2023-02-17

**Authors:** Faizal Hajamohideen, Noushath Shaffi, Mufti Mahmud, Karthikeyan Subramanian, Arwa Al Sariri, Viswan Vimbi, Abdelhamid Abdesselam, for the Alzheimer’s Disease Neuroimaging Initiative

**Affiliations:** 1 College of Computing and Information Sciences, University of Technology and Applied Sciences, Jamia Street, 311 Sohar, Sultanate of Oman; 2 Department of Computer Science, Nottingham Trent University, Clifton Lane, NG11 8NS Nottingham, UK; 3 Medical Technologies Innovation Facility, Nottingham Trent University, Clifton Lane, NG11 8NS Nottingham, UK; 4 Computing and Informatics Research Centre, Nottingham Trent University, Clifton Lane, NG11 8NS Nottingham, UK; 5 Department of Computer Science, Sultan Qaboos University, 123 Muscat, Sultanate of Oman

**Keywords:** MRI, Alzheimer’s disease, Classification, Siamese, Triplet-loss, Mild cognitive impairment

## Abstract

Alzheimer’s disease (AD) is a neurodegenerative disease that causes irreversible damage to several brain regions, including the hippocampus causing impairment in cognition, function, and behaviour. Early diagnosis of the disease will reduce the suffering of the patients and their family members. Towards this aim, in this paper, we propose a Siamese Convolutional Neural Network (SCNN) architecture that employs the triplet-loss function for the representation of input MRI images as *k*-dimensional embeddings. We used both pre-trained and non-pretrained CNNs to transform images into the embedding space. These embeddings are subsequently used for the 4-way classification of Alzheimer’s disease. The model efficacy was tested using the ADNI and OASIS datasets which produced an accuracy of 91.83% and 93.85%, respectively. Furthermore, obtained results are compared with similar methods proposed in the literature.

## Introduction

Neurological disorders (NLD) affect the central nervous system, such as the brain, spinal cord, nerves (cranial and peripheral), etc. Any minor disruption in the functionality of these will emerge as fatal physiological disorders. Alzheimer’s disease (AD) is one such NLD that has currently affected 55 million people worldwide according to the latest World Alzheimer report [1]. AD is the seventh leading cause of death worldwide which is an incurable, life-altering, and progressive neurodegenerative disease. The protein components inside the brain cells, also known as plaques and tangles, will undergo gradual degradation when afflicted by AD. Such impaired protein component will generate an enormous decline in cognitive abilities leading to severely degraded personal and social life [1, 2].

AD causes several discomforts that affect individuals wherein patients will have memory discomposure, behavioural disorderliness, and various other physical issues causing vision and mobility complications. The main bottleneck to early AD detection is the need for more of the public’s general knowledge about this disease. As a result, cognitive decline and related behaviours are often mistaken for phenomena associated with the normal ageing process and other psychiatric disorders. Also, factors such as remote locations, lack of skilled caregivers, and inaccessibility to experts and modern diagnostic tools will compound the suffering of patients [1] to the extent of interfering with their autonomy of daily and social life activities. Consequently, early AD detection is vital to minimise the patient’s suffering and the care-taking of family members.

**Fig. 1:**
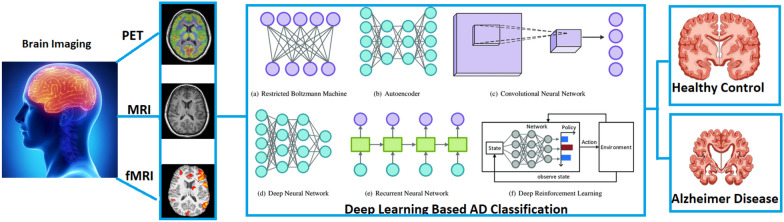
Block diagram showing the process of artificial intelligence-based Alzheimer’s disease classification

AD is diagnosed mainly by observation of patients’ symptoms, and sometimes it usually takes years to ensure the presence of the disease [1]. Nonetheless, advancements in diagnostic research have led to the discovery of several biomarkers (MRI, PET, CT, blood tests, etc.) that assist in early AD prediction. When coupled with Artificial Intelligence (AI) technologies, these biomarkers can assist doctors in accurate diagnosis and subsequent patient care. Machine learning (ML) classifiers have encompassed many healthcare sectors and have been very effective in AD classification [3–5]. Deep learning (DL) techniques especially have permeated the healthcare industry in the last several years for their ability to learn end-to-end models accurately. They can learn end-to-end models using compound data [6, 7]. This surge in the usage of DL methods has opened up possibilities for the accurate identification of neurological disorders in an unprecedented manner. When coupled with DL techniques, neuroimaging provides vital clues in the perception of brain activity and relevant disorders [8]. There are many effective computer-aided diagnosis (CAD) systems proposed for the prediction of AD using neuroimaging data, such as functional magnetic resonance imaging (fMRI), structural MRI (sMRI), and positron emission tomography (PET). The sMRI provides essential information such as brain white matter (WM), grey matter (GM), cortical thickness, and volumes that help measure the degeneration process affecting several brain regions resulting in AD. These high-definition image data and DL’s powerful modelling technique extract features that clinicians can elucidate for medical decision-making in complex AD disorders. Figure 1 demonstrates the typical process involved in DL-based techniques for AD classification.

The Siamese Convolutional Neural Network (SCNN) is a similarity-checking model that effectively encodes the input images in a way that the similarity between images of the same classes is higher than those belonging to different categories. The SCNN is proven to be an effective model for classifying data points whenever the samples in the dataset are limited or imbalanced [9, 10]. In [10], a new cost function framework was presented where the new triplet-loss function was introduced that leverages the concept of the Siamese network for optimal classification of facial images. In this paper, we employ the idea of SCNN along with the triplet-loss function for the four-way classification of AD. We utilised the popular VGG16 pre-trained architecture for the feature extraction of input MRI images to obtain the embeddings. The Siamese architecture will process these embeddings to transform the input space into an optimally clustered space where inter-class distance will be higher than intra-class distance. The model is developed and evaluated using the MRI images extracted from the Open Access Series of Imaging Studies (OASIS, www.oasis-brains.org) [11] and the Alzheimer Disease Neuroimaging Initiative (ADNI, https://adni.loni.usc.edu/) datasets. The proposed model does the four-way classification of MRI images where an input MRI image is classified as belonging to one of the four classes representing different stages of Alzheimer’s disease.

The work presented here is an extension of our previously published work [12]. This is our ongoing effort in researching Siamese architecture for AD detection. The extension covers the investigation of the approach using the ADNI dataset, provides a comparative study with existing methods, and additional experiments to corroborate the effectiveness of the applied techniques. Below are the salient contributions of this work:

1. We are leveraging VGG16 architecture in implementing Siamese architecture employing the triplet-loss function for four-way classification of AD.
2. The comparative study has been conducted with the methods from literature using the AI techniques in the four-way classification of AD.
3. The usage of the ADNI dataset in the performance evaluation of the proposed triplet-loss-based Siamese architecture.

The remainder of this paper is structured as follows: Sect. 2 presents the review of related literature. The proposed approach is presented in Sect. 3. The experimental results and associated analysis are presented in Sect. 4. The concluding remarks are drawn in Sect. 5.

## Related work

In recent years artificial intelligence (AI) has been applied in diverse problem domains to solve various challenging problems, including text classification [13–16], cyber security [17–20], neurological disease detection [12, 21–23] and management [24–29], elderly care [30, 31], fighting pandemic [32–38], and healthcare service delivery [39–41]. In particular, deep learning (DL) has attracted a lot of attention [6, 7]. The SCNN is proven to be an effective model for classifying data points whenever the samples in the dataset are limited or imbalanced [9, 10]. The concept of the Siamese network for similarity computation was initially proposed by Tiagman et al. [9] for efficient recognition of face images that resulted in a performance on par with human-level performance. Ever since, there have been numerous applications of SCNN in various domains of pattern recognition and computer vision [42]. In this section, we briefly outline some notable works using various Siamese CNN architecture in general medical analytics and specifically in AD classifications.

### Siamese in medical data analytics

The concept of Siamese similarity finding has also been extensively explored in diverse fields of medical analytics. This section briefly addresses some prominent applications of the Siamese concept in various medical areas.

Research studies have revealed that the karyotyping method uses Siamese networks with deep learning to analyse and order human chromosomes to diagnose various ailments automatically. In [43], authors have affirmed that it will be stimulating to train different Siamese networks to learn diverse behaviours collaboratively, increasing the accuracy of chromosome classifications.

In another study, Mohamed et al. [42] presented a Siamese network model using deep meta-learning to analyse the chest X-ray (CXR) images to classify the COVID-19-affected patients with 95.6% accuracy. The model has been trained with ten CXR samples and is planned to increase the CXR images with additional information like patient health history and location. The authors proved that their proposed Siamese model performs better than fine-tuned CNN models. For cellular disease identification, the categorisation of cellular assortment using Siamese for identifying the disease using multi-dimensional datasets and its challenges were presented by Benjamin et al. in [44]. The authors proposed a novel framework that decreases the dimensionality using a Siamese neural network. This Siamese network is then trained using the triplet-loss function, which allows it to train hundreds of cells linearly.

Kelwin et al. [45] developed a deep Siamese learning model to find cervical cancer using the patient’s biopsy and individual medical records. The proposed model in this paper focuses mainly on reducing the dimensionality of data and works in low-dimensional space. It gives more accuracy in prediction than existing models, such as denoising autoencoders. Research in [46] used Siamese networks to find the similarity of gene expression patterns between two compounds to identify the structural match of drugs. The proposed model is more successful at identifying the chemical compounds on the drugs than other existing models, with a Pearson correlation of 0.518. The authors use this model to reduce the search space for new drug discoveries. Yet another research [47] used the concept of Siamese in Spinal metastasis detection, which is mainly used to find metastatic cancer on the spine.

Early detection of diabetic retinopathy (DR) using an automatic diagnostic system instead of a manual process helps avoid early blindness. To increase the diagnosis accuracy, Xianglong et al. [48] proposed a Siamese-like architecture to train conventional neural networks (CNN) using binocular fundus images. This model provides a higher area under the curve (AUC) accuracy of 0.951 than the existing monocular model, which shows a lower AUC accuracy of 0.940. In another study in a similar field, Yu-An et al. [49] proposed a learning model trained on a subset of the diabetic retinopathy (DR) fundus image dataset with binary image representations using deep Siamese CNN. The authors proved that their approach required less supervision and reduced the computational expense of medial image machine learning. Shreyas et al. [50] proposed a hybrid model combining stacked bidirectional long short-term memory (LSTM) and LSTMs with Siamese network architecture. It classifies brain fibre tracts into meaningful clusters with high accuracy using high-accuracy tractography data as an input. The experimental results proved that the accuracy is lower in inter-brain images than in intra-brain pictures due to the possibility of varying size and shape.

### Siamese in Alzheimer’s disease prediction

Here, we review papers that dealt with Alzheimer’s disease classification using general machine learning algorithms, including Siamese.

In [51], the deep Siamese neural network was used to enhance the discriminatory feature of whole-brain volumetric asymmetry. This paper demonstrated the performance to be on par with the model that utilises whole-brain MRI images. In [52], the authors have used the pre-trained VGG-16 model to classify AD using the OASIS dataset. They have achieved a test accuracy of 99.05%. In [53], authors have proposed multi-modal data for training to predict the evolution of the disease. This achieved an accuracy of 92.5% on the Alzheimer’s Disease Neuroimaging Initiative (ADNI) dataset that was curated to consider the baseline and 12-month MRI. Mehmood et al. [54] have proposed a transfer learning-based CNN classification to diagnose the early stage of AD using the ADNI dataset. Authors have provided a 2-way classification (AD vs CN & EMCI vs LMCI) using this method and obtained an accuracy of 98.37% and 83.72%, respectively. In [55], authors have applied CNN to classify AD using MRI images collected using the ADNI dataset. The algorithm applies specifically preprocessing to the images of MRI to aid in the efficient diagnosis of AD. In another study, [56], authors have used the popular AlexNet model for the feature extraction from the brain MRI images for subsequent classification by popular tools such as Random Forest (RF), Support Vector Machine (SVM), k-Nearest Neighbour (KNN), etc. The proposed approach resulted in good accuracy in the classification of AD disease. Arifa et al. [57] propose a hybrid approach to train a deep neural network that combines the features from MRI and EEG signals. The main idea of this approach is to consider multi-modal data in training the classifier. Chitradevi et al. [58] presented an approach to segment the cerebral sub-regions for efficient AD classification. The segmented output is fed to machine learning classifiers for diagnosing AD, which resulted in 98% accuracy using the Grey-Wolf optimisation approach.

Based on this recent brief review of the research, we can conclude that:

1. Siamese deep learning architecture is widely used in medical data analysis. This prompted us to evaluate the SCNN using the Triplet-loss function for AD classification.
2. Many works have been reported that leverage the CNN architecture for AD classification purposes either by using pre-trained models [52, 56] or a minimalist non-pretrained CNN model [55]. These models have obtained satisfactory results.
3. Not many works reported the use of Siamese in the recent past for the classification of AD except for a few works [51–53]. Although there has been reported use of SCNN for the classification of AD, employing the triplet-loss function for learning the underlying brain MRI for optimal separation of Alzheimer’s classes are not done to the best of our knowledge.

We overcome the above-mentioned gap by employing the pre-trained VGG16 model as an encoder and transforming the MRI images into efficient lower-dimensional embeddings. These embeddings were later trained using the triplet-loss function for the four-way classification of Alzheimer’s Disease using the OASIS and ADNI datasets. To compare the performance of the pre-trained model, we have also utilised the non-pretrained simplistic CNN model. We believe this work will serve as a prelude for many works that exploit the benefit of triplet-loss coupled with various DeepNet architectures in the literature.

## Methods

In this section, we present the building blocks of the Siamese CNN using the triplet-loss function to classify AD.

### Siamese CNN architecture

**Fig. 2:**
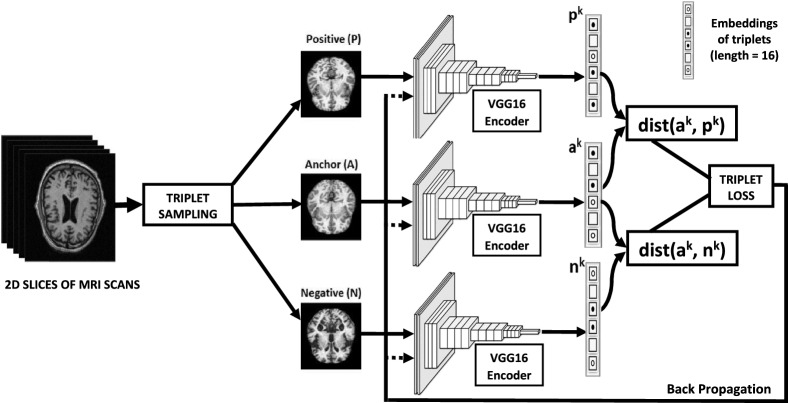
The architecture of the Siamese network

The Siamese Convolutional Neural Network (SCNN) has two or more identical sub-networks working together to generate feature vectors for input images enabling similarity score computation. The whole process is shown in Fig. 2. This process aims to learn a similarity model such that images of the same class will have embedding that contrasts significantly when an image of different classes is fed. From the batch of input images, three images (triplets) will be randomly sampled: where two images, anchor (A) and positive (P), will be from the same class, whereas the third image, negative (N), will be from a different class. The SCNN aims to produce embeddings for every image in the triplet: A, P, and N so that the distance from the anchor image to the positive image becomes closer than the anchor to the negative image as depicted in Fig. 2.

The triplets (*A*, *P*, *N*) will be fed through similarly weighted deep ConvNet encoders, which transform the triplets into an embedding space $F_w (I^{a})$, $F_w (I^{p})$, $F_w (I^{n})$ through flattening of the last layer of VGG16. In this embedding space, images belonging to the different classes are expected to form tightly coupled well-separated clusters. It is to be noted that the SCNN, although depicted as having separate branches, essentially has a single ConvNet encoder that sequentially extracts features of *A*, *P*, and *N* images. The L2 distance metric can be used to measure the distance between (A, P) and (A, N) pairs as $d(A, P) = |\,| F_w(I^{a}) - F_w (I^{p}) |\,|$ and $d(A, N) = |\,| F_w (I^{a}) - F_w(I^{n}) |\,|$.

The triplet-loss function is used at this stage using the *d*(*A*, *P*) and *d*(*A*, *N*) to compute the loss of the learned model. Finally, The similarity score is transformed into a range of 0 to 1 through the cosine similarity measure. the similarity of (*A*, *P*) is expected to be larger than that of (*A*, *N*).

### Triplet-loss function

In [10], a new framework was proposed where the triplet-loss function was introduced that leverages the concept of the Siamese network for optimal classification of facial images. It performed way better than the conventional contrastive loss function [59] adopted in Siamese learning.

This is a loss function-based distance measure that needs three inputs. Given *A*, *P*, and *N* images:

1 $$\begin{aligned} \mathcal {L}(A,P,N) = {\text{max}} (d(A,P) - d(A,N)+\alpha , 0). \end{aligned}$$

If there are *m* training triplet images, the overall cost function for the SCNN would be:

2 $$\begin{aligned} \mathcal {J}(L(A, P, N)) = \sum _{i=1}^{m} \mathcal {L}(A_i, P_i, N_i). \end{aligned}$$

It is easy to satisfy the constraint $d(A, P)-d(A, N)+\alpha$. Thus, hard triplets are chosen such that $d(A, P)\approx d(A, N)$. The ensuing step of Gradient Descent will minimise the loss function such that $d(x^{(i)}, x^{(j)})$ is less for identical pairs, typically for* A* and* P*, and more significant for* A* and* N*.

### The ConvNet encoders

We tested the proposed model’s efficacy by using both pre-trained and non-trained CNN architectures. This will help us determine the SCNN model’s performance efficacy under different scenarios, such as the applicability of a very deep ConvNet encoder and its impact on the embeddings, the influence of pre-trained weights, etc.

#### The non-trained CNN

We considered a simplistic non-trained CNN architecture having three convolutional layers, two pooling layers, and 2 fully connected (FC) or dense layers. The CNN used has a configuration of $64C7-MP2-64C3-MP2-128C3-FC1024-FC4$; where *nCj* denotes *n* convolutional layer with $j \times j$ filters, *MPk* indicates a maxpooling layer with $k \times k$ kernel, and *FCn* indicates an FC layer with *n* neurons. In this manner, each triplet (*A*,* P*,* N*) is transformed into a *k*-dimensional embedding/feature vector.

**Fig. 3:**
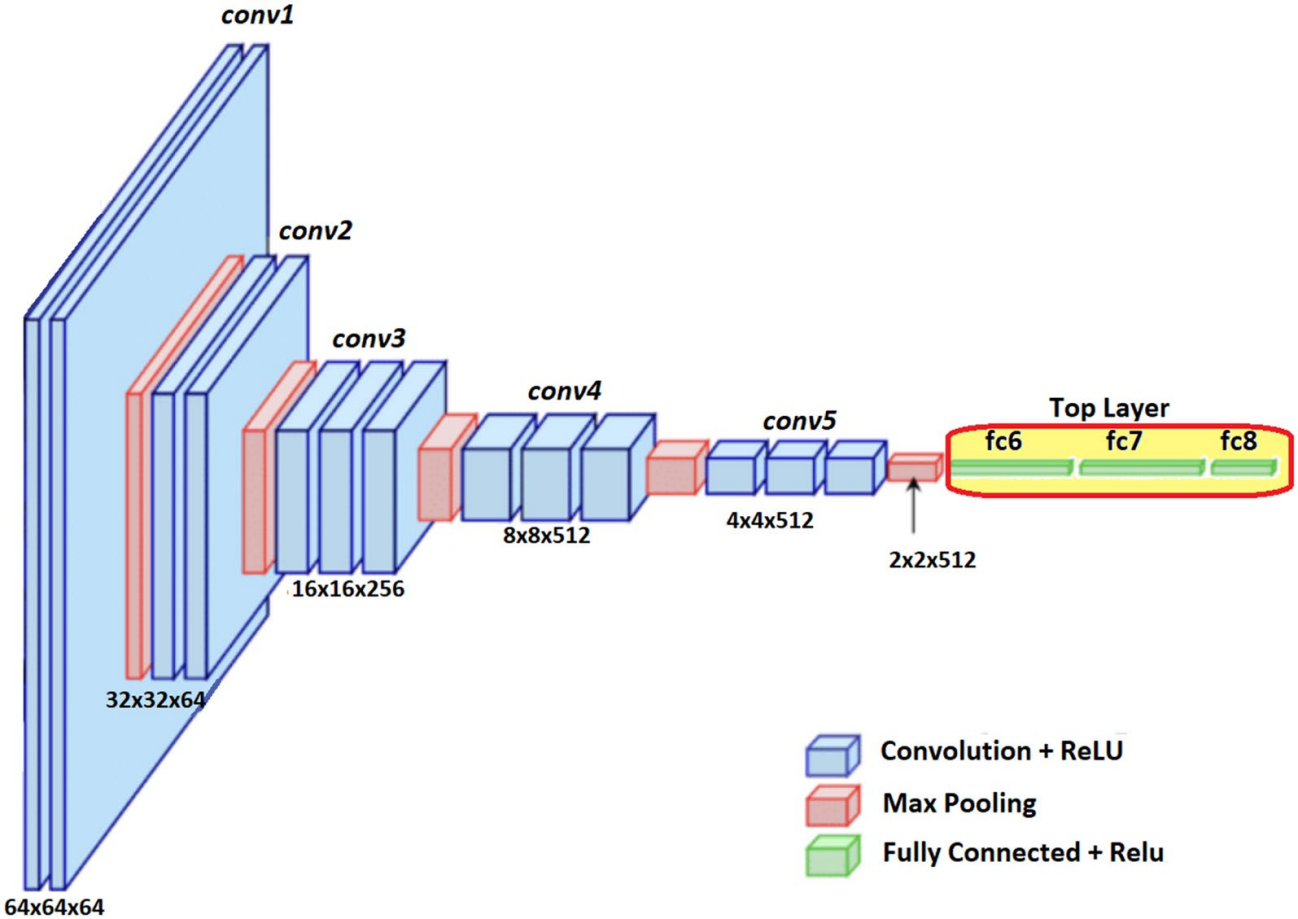
The architecture of the VGG16 model used as ConvNet encoder

#### The pre-trained VGG16 model

The VGG16 model [60] is one of the popular pre-trained models used in computer vision problems [42]. Motivated by its success, this architecture has been used in our proposed work as a feature extractor for Siamese architecture and as a traditional classifier. The VGG16 architecture is shown in Fig. 3. The three dense layers, known as the top layer, will be replaced by one or two dense layers catering to our requirements. When VGG16 is used as a ConvNet encoder, the Siamese model will use a single dense layer to extract a 1-dimensional feature vector of length *k*. When used as a classifier, the top layer will be replaced by two dense layers of dimensions 256 and 4, respectively.

**Fig. 4:**
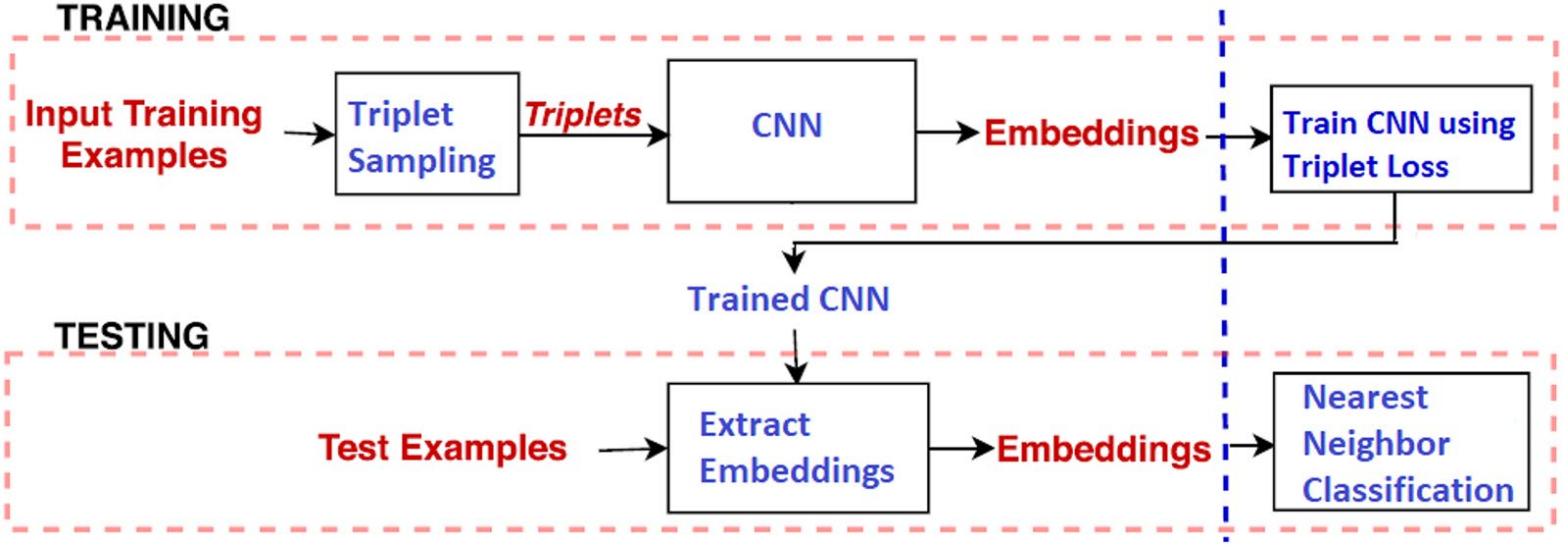
The training and testing phases

The overall process involved in the triplet-loss-based SCNN training and subsequent classification of AD can be outlined in Fig. 4.

## Experimental results

This section presents a series of experiments to corroborate Siamese architecture’s efficacy using two publicly available databases: the Open Access Series of Imaging Studies (OASIS) [11] and the Alzheimer’s Disease Neuroimaging Initiative (ADNI) [61] dataset.

The OASIS-3 dataset [11] categorises the brain MRI images into four classes based on clinical dementia rating (CDR). There were 755 participants with no history of dementia (CN), and 622 individuals participated with various stages of dementia in the age group ranging from 42–95 years. Four CDR scores are considered in forming this database: 0, 0.5, 1.0, and 2.0. The CDR score of 0 indicates *No Dementia*, 0.5 indicates *Very Mild Dementia*, 1.0 indicates *Mild-Dementia*, and 2.0 indicates *Moderate Dementia*. The number of samples in CDR-0, CDR-0.5, CDR-1, and CDR-2.0 is 3200, 2240, 896, and 64 images. Using this dataset, the 4-way classification can be performed based on the CDR rating (CDR-0 vs CDR-0.5 vs CDR-1 vs CDR-2).

**Fig. 5:**
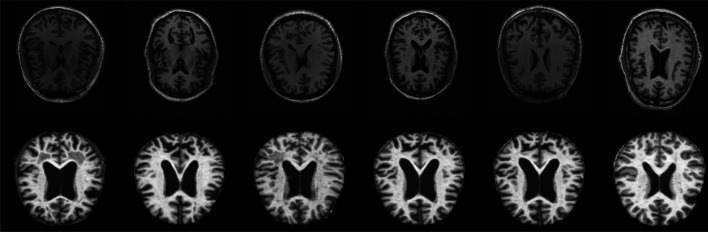
Sample images from datasets: ADNI (top-row) and OASIS (bottom-row)

The ADNI dataset was acquired from three phases: ADNI-1, ADNI GO and ADNI 2. The study group’s ages ranged from 50–65 years. We used the MPRAGE baseline 1.5T T1-weighted MRI images in the axial plane with a pixel dimension of $2048 \times 2048$ from the sagittal slices. The images were resized to $64 \times 64$ in our experiments. The dataset contains images from 162 participants, out of which 37 are AD patients, 12 are cognitively normal (CN), 53 are considered to have mild-cognitive impairment (MCI), and 60 are in the Early MCI stage (EMCI). The collected samples include 739 MRIs having AD, 157 MRIs representing CN cases, 717 images having mild cognitive impairment (MCI), and 1446 having early MCI (EMCI) conditions. Using this dataset, the 4-way classification of an MRI image is done (AD vs MCI vs EMCI vs CN). Each participant has multiple samples collected at various intervals of time. Sample images from these two datasets are shown in Fig. 5. As OASIS is already preprocessed (such as bias correction, skull-stripping, etc.), we did not perform any additional preprocessing for this dataset. Also, as deep learning models are known to learn in an end-to-end manner, it does not necessitate a preprocessing step compared to conventional machine learning models. Hence, we have not performed any preprocessing of these images. The images used in the experiments were in grayscale TIFF format.

Three different performance metrics are used, namely, *Accuracy*, *Specificity*, and *Sensitivity*. The accuracy (Eq. 3) is a ratio of correctly classified MRIs to the total MRIs available in the dataset. Sensitivity (or Recall) refers to the number of times the model correctly predicts the MRI as a positive case out of total positive cases (Eq. 4). The Sensitivity is also called *True Positive Rate (TPR)*, using which *False Negative Rate (FNR)* can be inferred as *1-sensitivity*. The Specificity measures the model’s ability to rightly classify an MRI with a negative case among the total number of negative cases (Eq. 5). Specificity is also known as *True Negative Rate (TNR)*. This measure also helps to discover the *false positive rate (FPR)* as *1-specificity*. It is to be noted that all reported metrics are averaged with the one vs all strategy.

3 $$\begin{aligned}{} & {} {\text{Accuracy}} = \frac{\rm{TP + TN}}{\rm{TP + TN + FP + FN}}, \end{aligned}$$

4 $$\begin{aligned}{} & {} {\text{Sensitivity}} = \frac{\rm{TP}}{\rm{TP+FN}}, \end{aligned}$$

5 $$\begin{aligned}{} & {} {\text{Specificity}} = \frac{\rm{TN}}{{\rm TN} + {\rm FP}}. \end{aligned}$$

The hyperparameters used in our experimentation are shown in Table 1. Unless mentioned explicitly, the values of the hyperparameters referred to in this article conform to this table.

**Table 1:** Hyperparameter values used in the architecture

Hyperparameter	Value
Embedding size	16
Loss function	Triplet-loss (Refer Eq. 2)
Batch size	128
Epochs	250
Activation function	ReLU
Optimizer	Adam
Learning rate	0.00001

**Fig. 6:**
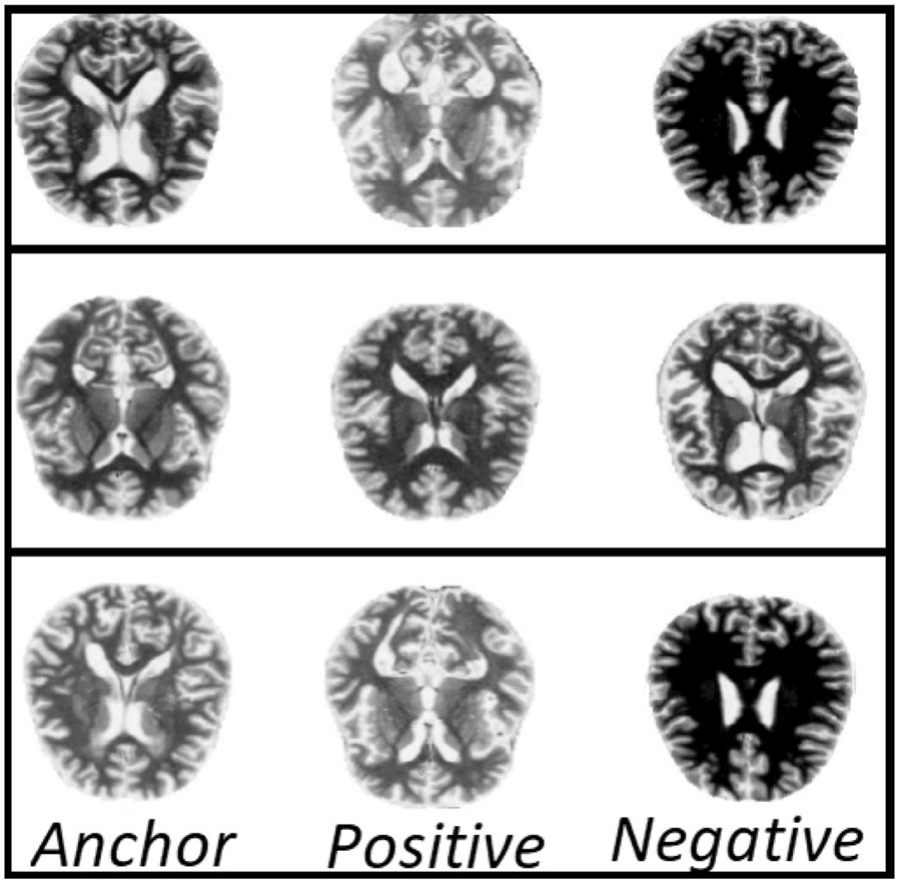
Examples of triplets formation

The batch size used in our experiment was 128. We chose 64 hard triplets, and the remaining 64 were chosen randomly. We randomly selected 200 samples from our dataset for every batch and extracted embeddings using the VGG16 ConvNet encoder. We then computed the distance vectors as $d(A, P)-d(A, N)$ and chose the hard triplets such that $d(A, P) \approx d(A, N)$. The train and test samples are drawn in this manner for mentioned number of epochs (see Table 1) in order to train the Siamese architecture. A few sample triplets chosen in this manner are shown in Fig. 6.

**Fig. 7:**
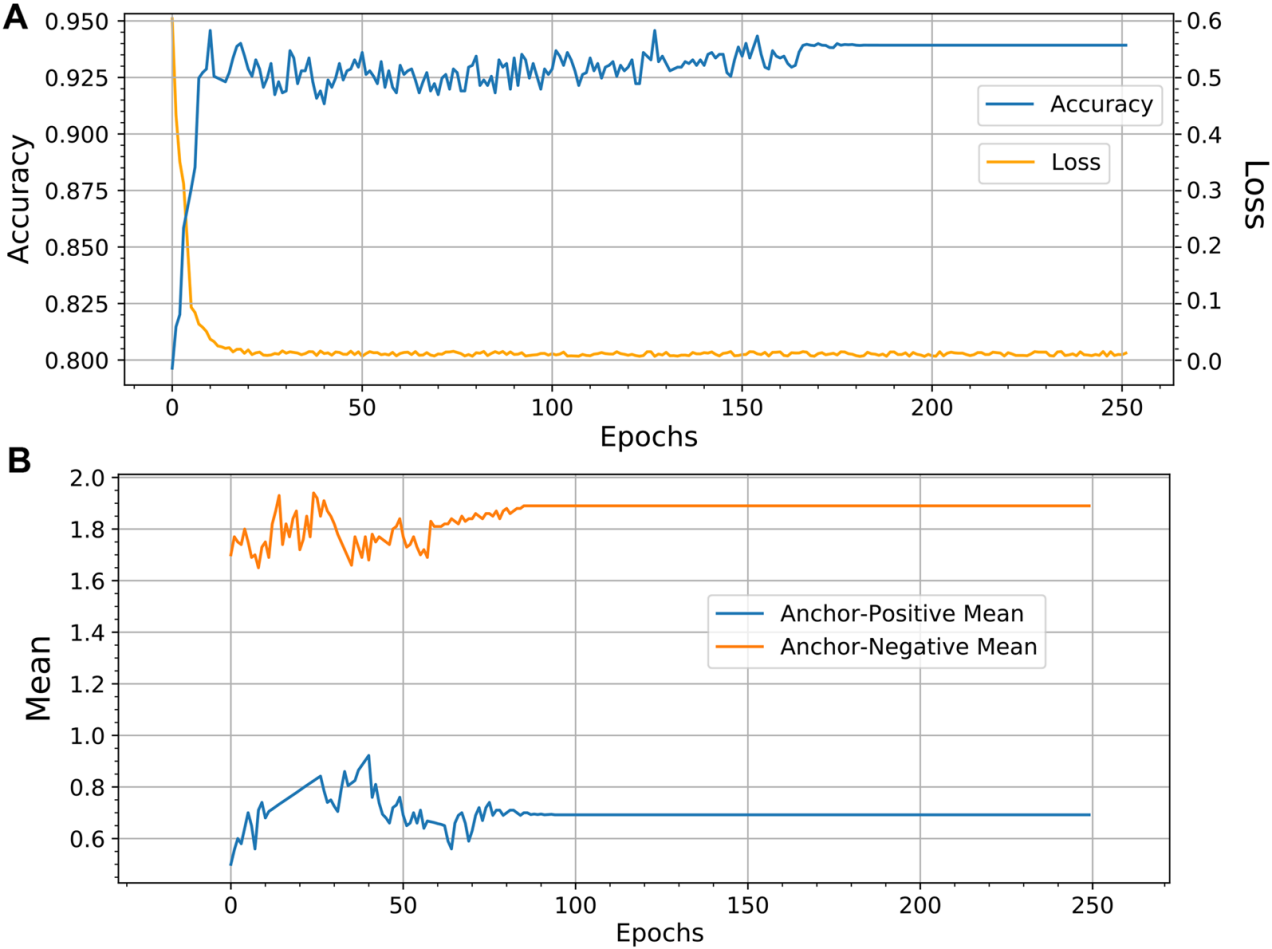
Model learning for 250 epochs (**A**) along with the means of the anchor-positive and anchor-negative learning (**B**)

One of the influential hyperparameters in this study is the size of the embedding feature vector. To determine this, we ran a series of experiments by varying the length of the embedding size from $2^{3}$ to $2^{7}$ in steps of power of 2. We empirically fixed the embedding length to be 16. The experiments determined that the length of the embedding vector did not impact the model’s convergence or the loss value. The value $k=16$ yielded optimal performance; hence, we fixed this value for embedding size for the rest of our experiments.

The implementation was carried out in Python 3.7.10 using Keras deep learning API. The model was trained using Intel i7 1.6 GHz CPU having 16 GB RAM supported by a GPU card (NVidia GeForce MX330). We also utilised *EarlyStopping* Keras callback to avoid overfitting the model.

The model was trained for 250 epochs without the *EarlyStopping* Keras callback to get a holistic view of the overall similarity model learning using the Siamese architecture. The training loss and validation accuracy plot are shown in Fig. 7A. We can observe that training loss converged around the 15th epoch, but the validation accuracy fluctuated before becoming steadier around the 170th epoch. This is because the validation accuracy represents the accuracy obtained for similarity learning using a batch of images during each training epoch. The model fluctuates with test accuracy on random images before learning useful patterns around the 170th epoch. Figure 7B shows the relation between the anchor-positive (AP) score and anchor-negative (AN) score. The AP score is the mean distance between all the anchor and positive images; the AN score is the mean distance computed between all anchor-negative pairs. It was calculated during the initial training of the model for 250 epochs. The model’s ability to distinguish similar and dissimilar pairs of images is evident in the mean distance computed at each epoch of the experiment.

**Fig. 8:**
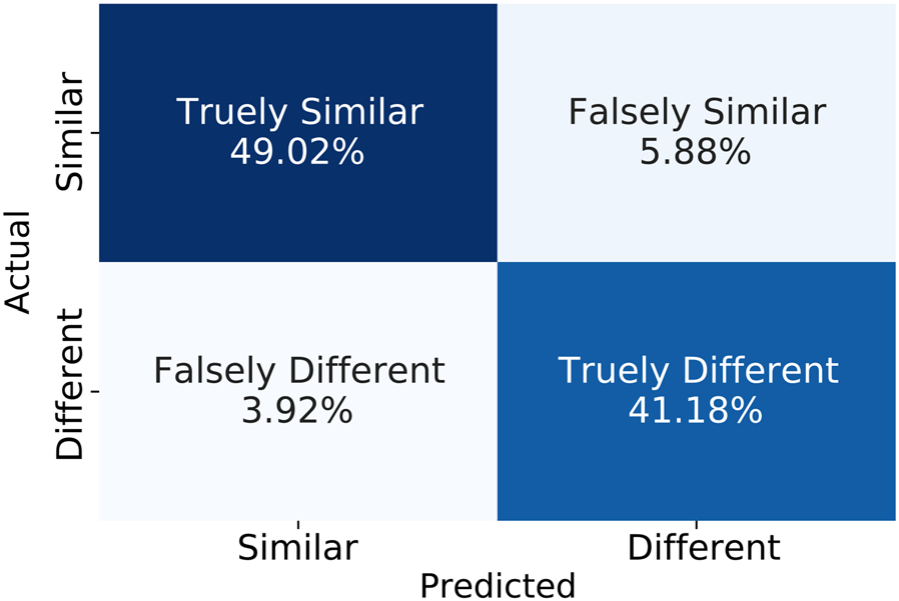
Confusion matrix of the similarity learning

After this similarity model learning, we tested with a batch of test triplets. We measured the distance between the anchor and positive images, and thresholding was carried out to classify them as belonging to the same or different classes. We did a similar computation between the anchor and negative images. Figure 8 depicts the confusion matrix thus obtained after evaluating with the true labels. This initial set of experiments was carried out to test the model convergence in classifying AD MRI images.

**Table 2:** Results of proposed models on the OASIS dataset

Models	Sensitivity	Specificity	1-Sensitivity	1-Specificity	Accuracy
Siamese CNN	0.9390	0.9561	0.0609	0.0439	0.9385
Siamese CNN with VGG16	0.9216	0.970	0.0784	0.029	0.9217
VGG16 (as classifier)	0.903	0.960	0.186	0.129	0.9020

**Table 3:** Results of proposed models on the ADNI dataset

Models	Sensitivity	Specificity	1-Sensitivity	1-Specificity	Accuracy
Siamese CNN	0.9179	0.9383	0.0821	0.0670	0.9183
Siamese CNN with VGG16	0.950	0.853	0.146	0.0490	0.8531
VGG16 (as classifier)	0.963	0.891	0.108	0.0390	0.8900

**Table 4:** Comparative study with existing methods

Method	Dataset	Accuracy (%)	Remark
Ensemble of deep neural network [62]	OASIS	93.18	4-way classification. Custom CNNs used in the Ensemble
Transfer Learning using AlexNet [63]	OASIS	92.85	4-way classification
Ensemble of 2 SVMs [64]	OASIS	69.1	For binary classification accuracy 93.2%
Ensemble of 5 transfer learning [64]	OASIS	70.6	For binary classification accuracy 90.2%
SVM [65]	OASIS	77.0	For binary classification accuracy 97.0%
Multi-kernel SVM [66]	ADNI	93.2	This work discusses various ML techniques
SVM and CNN [67]	ADNI	96.0	3-way classification for predetection of AD through AD vs MCI vs CN.
3D Ensemble of DenseNet [8]	ADNI	83.33	4-way classification
Proposed model	OASIS	**93.85**	4-way classification
Proposed model	ADNI	**91.83**	4-way classification

Bold indicates the value obtained for proposed model

**Fig. 9:**
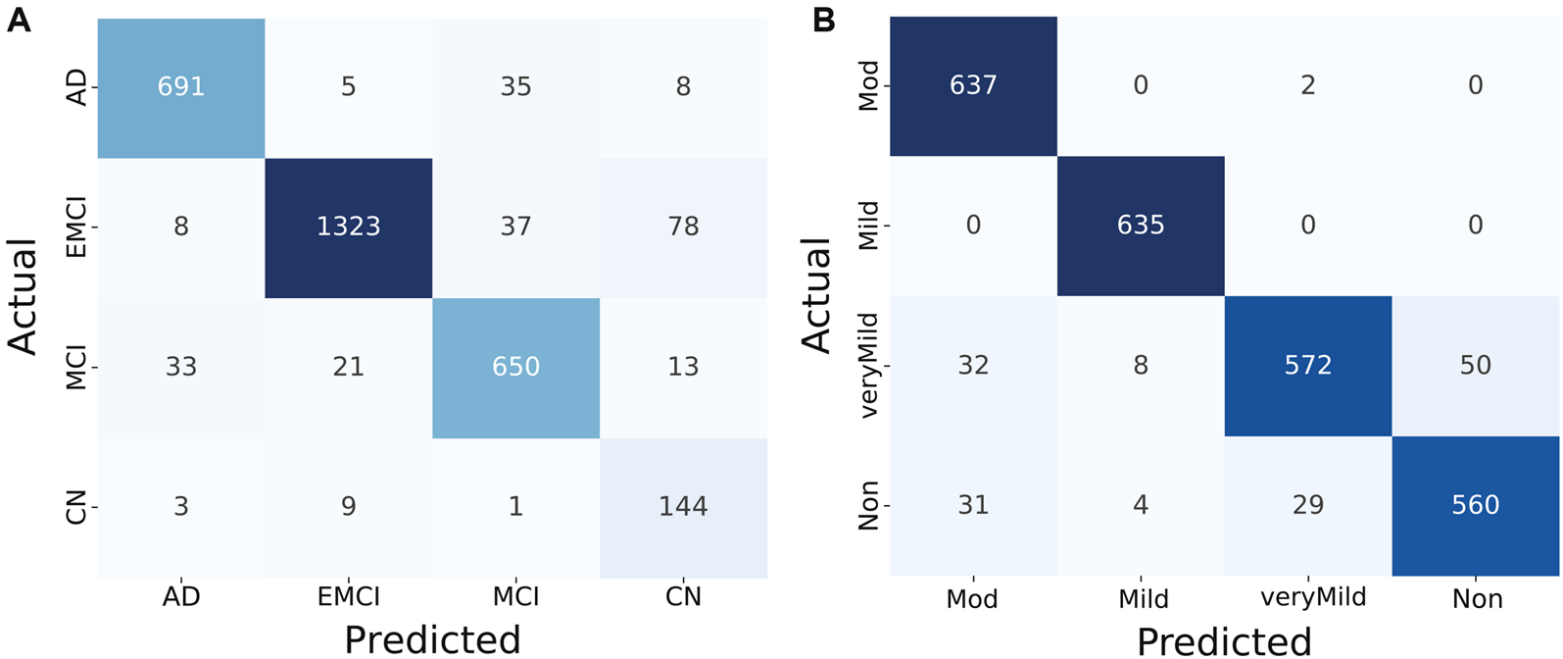
Confusion matrices obtained using the ADNI dataset (**A**) and the OASIS dataset (**B**)

The experimental findings of the proposed model are shown in Tables 2 and 3. The confusion matrices for the best-performing models from this experiment are shown in Fig. 9. Some important observations from this experiment are:

1. The proposed model for the 4-way classification of AD achieves overall good recognition accuracy considering the limited samples and class imbalance in the dataset we considered. This is the true benefit of using Siamese architecture. The usage of the triplet-loss function further enhances the class separability among the four classes where samples of some classes have finer distinctive features (for instance, CDR-0.5 and CDR-1.0, CN and EMCI, AD and MCI).
2. Although the deep ConvNet models are known to perform well, their performance on test data is lesser than the basic ConvNet encoder we proposed. This may be due to using pre-trained weights in the frozen layers. Setting those layers to a trainable option with an empirically determined learning rate would probably yield the best results [68].
3. Inspired by the success of the VGG16 model in solving many computer vision problems, we leveraged the pre-trained VGG16 model as a traditional classifier for the 4-way classification of the AD. Firstly, the input samples were resized to $64 \times 64 \times 3$ to be compatible with its architecture. The VGG16 model was trained using the ImageNet weights and without the top layer. We added two dense layers, the final layer being the output 4-neuron layer, to classify the AD. The results of this model are not better than the proposed Siamese-based one. There could be many reasons for this, but the main point here is that we have insufficient samples to train the model. The reasonable accuracy it obtained was probably due to the immense training the model initially underwent for the ImageNet competition. Those convolutional kernels could have contributed to the accuracy. This gives further scope for future avenues.

Finally, Table 4 compares the results obtained with existing models based on machine learning and deep learning techniques. The following main observations are made from this comparative study:

1. The proposed models achieved better or comparable accuracy with most existing methods. In addition, there is an inherent advantage of better generalisation with limited training samples in the case of proposed models.
2. Two of the existing methods [66, 67] obtained higher accuracy than the proposed method, emphasising that there is scope for improvement with the proposed model. However, the existing approaches utilised the ADNI dataset [67] which differs from the collection we considered in this study. Similar reasoning holds good for the approach proposed by Ruiz et al. [8] that has lower recognition rate compared to other models.

## Conclusion

Inspired by the success of Siamese architecture in the diagnosis of Covid-19 using chest X-ray images [42], in this study, we presented the applicability of Siamese architecture using the triplet-loss function for the 4-way classification of AD using the MRI images. The Alzheimer’s disease classification falls under the data-scarce domain, where getting sufficient training samples to train a conventional neural network adequately may not be practical. The work presented in this article demonstrated that a Deep Siamese architecture could alleviate this limited data problem typical in most medical domains. We used the triplet-loss function to calibrate the Siamese architecture in contrast to the contrastive loss function applied [42]. The model was tested using the OASIS and ADNI datasets, resulting in an accuracy of 93.85% and 91.83%, respectively.

In the future, we can do several extensions from the proposed work; a few prominent ones are mentioned below:

1. We utilised the VGG16 deep architecture to obtain the input samples’ embeddings. One can conduct a thorough performance evaluation using various pre-trained networks such as GoogleNet, AlexNet, ResNet and its variants, etc., to determine how well the Siamese model generalises in the 4-way classification of AD.
2. We have used axial planes of MRI images from the ADNI dataset. It would be interesting to see the fusion of information from sagittal and coronal planes in modelling the similarity.

The Siamese architecture using the triplet-loss function is yet to see broader applicability in AD classification. Our work presented here will serve as good reference material for many such results in the future.

## Data Availability

This work used two datasets (ADNI and OASIS) which are available in the public domain. The ADNI dataset is available at https://adni.loni.usc.edu/ and the OASIS dataset is available at https://www.oasis-brains.org/. The developed methods can be obtained by contacting the authors.
